# Enzyme immobilization: an overview on techniques and support materials

**DOI:** 10.1007/s13205-012-0071-7

**Published:** 2012-06-06

**Authors:** Sumitra Datta, L. Rene Christena, Yamuna Rani Sriramulu Rajaram

**Affiliations:** School of Chemical and Biotechnology, Shanmuga Arts, Science, Technology and Research Academy (SASTRA) University, Tirumalaisamudram, Thanjavur, 613401 Tamilnadu India

**Keywords:** Enzyme immobilization, Techniques, Supports, Applications

## Abstract

The current demands of the world’s biotechnological industries are enhancement in enzyme productivity and development of novel techniques for increasing their shelf life. These requirements are inevitable to facilitate large-scale and economic formulation. Enzyme immobilization provides an excellent base for increasing availability of enzyme to the substrate with greater turnover over a considerable period of time. Several natural and synthetic supports have been assessed for their efficiency for enzyme immobilization. Nowadays, immobilized enzymes are preferred over their free counterpart due to their prolonged availability that curtails redundant downstream and purification processes. Future investigations should endeavor at adopting logistic and sensible entrapment techniques along with innovatively modified supports to improve the state of enzyme immobilization and provide new perspectives to the industrial sector.

## Introduction

Enzymes or ‘biocatalysts’ are remarkable discovery in the field of bioprocess technology. Biocatalysis has been widely accepted in diverse sectors owing to their ease of production, substrate specificity and green chemistry. However, for large extent commercialization of these bio-derived catalysts, their reusability factor becomes mandatory, failing which they would no longer be economic. Maintenance of their structural stability during any biochemical reaction is highly challenging. Consequently, immobilized enzymes with functional efficiency and enhanced reproducibility are used as alternatives in spite of their expensiveness. Immobilized biocatalysts can either be enzymes or whole cells (Kawaguti et al. 2006). Enzyme immobilization is confinement of enzyme to a phase (matrix/support) different from the one for substrates and products. Inert polymers and inorganic materials are usually used as carrier matrices. Apart from being affordable, an ideal matrix must encompass characteristics like inertness, physical strength, stability, regenerability, ability to increase enzyme specificity/activity and reduce product inhibition, nonspecific adsorption and microbial contamination (Singh 2009). Immobilization generates continuous economic operations, automation, high investment/capacity ratio and recovery of product with greater purity (D’Souza 1998). Several methods are used for immobilization and various factors influence the performance of immobilized enzymes (Table 1). Adsorption/carrier-binding method uses water-insoluble carriers such as polysaccharide derivatives, synthetic polymers and glass (Al-Adhami et al. 2002; Rosa et al. 2002; Wu and Lia 2008; Cordeiro et al. 2011). In cross-linking/covalent method, bi/multifunctional reagents such as glutaraldehyde, bisdiazobenzidine and hexamethylene diisocyanate are used (Lee et al. 2006; Singh 2009). Polymers like collagen, cellulose and κ-carrageenan are employed by entrapment method, while the membrane confinement method includes formulation of liposomes and microcapsules (Katwa et al. 1981; Wang and Hettwer 1982; Mislovicová et al. 2004; Hilal et al. 2006; Tümtürk et al. 2007; Rochefort et al. 2008; Jegannathan et al. 2010; Chen et al. 2011a, b; Klein et al. 2011).

**Table 1:** Factors influencing performance of immobilized enzymes (Cao 2006)

Factors	Implications of immobilization
Hydrophobic partition	Enhancement of reaction rate of hydrophobic substrate
Microenvironment of carrier	Hydrophobic nature stabilizes enzyme
Multipoint attachment of carrier	Enhancement of enzyme thermal stability
Spacer or arm of various types of immobilized enzymes	Prevents enzyme deactivation
Diffusion constraints	Enzyme activity decreases and stability increases
Presence of substrates or inhibitors	Higher activity retention
Physical post-treatments	Improvement of enzyme performance
Different binding mode	Activity and stability can be affected
Physical structure of the carrier such as pore size	Activity retention was often pore-size dependent
Physical nature of the carrier	Carriers with large pore size mitigate diffusion limitation, leading to higher activity retention

This article reviews the existing techniques used for immobilization along with providing insights into the recent developments for each of them. We have tried to throw light on significant modifications with respect to the techniques and innovative support materials employed for immobilization of biocatalysts that have potential implication on future enzyme market.

## Different techniques used for immobilization

### Adsorption

Enzyme adsorption results from hydrophobic interactions and salt linkages where either the support is bathed in enzyme for physical adsorption or the enzyme is dried on electrode surfaces. Adsorbed enzymes are shielded from aggregation, proteolysis and interaction with hydrophobic interfaces (Spahn and Minteer 2008). Researchers have used eco-friendly supports like coconut fibers having good water-holding capacity and high cation exchange property; microcrystalline cellulose with irreversible binding capacity; kaolin with high enzyme retainability by chemical acetylation; and micro/mesoporous materials having thiol functionalized, large surface area ideally suited for reduction and oxidation reactions (Dey et al. 2002; Hernández et al. 2007; Karagulyan et al. 2008; Brígida et al. 2010; Mitchell and Ramírez 2011; Huang et al. 2011). Silanized molecular sieves have also been successfully used as supports for enzyme adsorption owing to the presence of silanols on pore walls that facilitate enzyme immobilization by hydrogen bonding (Diaz and Balkus 1996). Various chemical modifications of the currently used supports would definitely help in better immobilization. Water activity profiles of lipase adsorbed using polypropylene-based hydrophobic granules/Accurel EP-100 has been reported (Persson et al. 2000). It would be important to note that Accurel with smaller particle sizes increases reaction rates and enantiomeric ratios during biocatalyzation (Sabbani et al. 2006).

For better process control and economic production, *Yarrowia lipolytica* lipase was immobilized on octyl-agarose and octadecyl-sepabeads supports by physical adsorption that resulted in higher yields and greater (tenfold) stability than that of free lipase. This was accounted by the hydrophobicity of octadecyl-sepabeads that enhances affinity between the enzyme and support (Cunha et al. 2008). *Candida rugosa* lipase adsorbed on biodegradable poly (3-hydroxybutyrate-co-hydroxyvalerate) showed 94 % residual activity after 4 h at 50 °C and reusability till 12 cycles (Cabrera-Padilla et al. 2011). These supports were preferred because they are less tough and crystalline than polyhydroxybutyrate. 1, 4-Butenediol diglycidyl ether-activated byssus threads have been suitable basement for urease that increased pH stability and retained 50 % enzyme activity under dried conditions (Mishra et al. 2011). Eco-friendly supports of biological origin not only prevent cropping up of ethical issues, but also cut down the production costs. Of late, biocompatible mesoporous silica nanoparticles (MSNs) supports have been used for biocatalysis in energy applications owing to their long-term durability and efficiency (Popat et al. 2011).

### Covalent binding

Covalent association of enzymes to supports occurs owing to their side chain amino acids like arginine, aspartic acid, histidine and degree of reactivity based on different functional groups like imidazole, indolyl, phenolic hydroxyl, etc. (D’Souza 1998; Singh 2009). Peptide-modified surfaces when used for enzyme linkage results in higher specific activity and stability with controlled protein orientation (Fu et al. 2011). Cyanogen bromide (CNBr)-agarose and CNBr-activated-Sepharose containing carbohydrate moiety and glutaraldehyde as a spacer arm have imparted thermal stability to covalently bound enzymes (Hsieh et al. 2000; Cunha et al. 2008). Highly stable and hyperactive biocatalysts have been reported by covalent binding of enzymes to silica gel carriers modified by silanization with elimination of unreacted aldehyde groups and to SBA-15 supports containing cage-like pores lined by Si–F moieties (Lee et al. 2006; Szymańska et al. 2009). Increase in half-life and thermal stability of enzymes has been achieved by covalent coupling with different supports like mesoporous silica, chitosan, etc. (Hsieh et al. 2000; Ispas et al. 2009). Cross-linking of enzymes to electrospun nanofibers has shown greater residual activity due to increased surface area and porosity. Use of such nanodiametric supports have brought a turning point in the field of biocatalyst immobilization (Wu et al. 2005; Kim et al. 2006; Ren et al. 2006; Li et al. 2007; Huang et al. 2008; Sakai et al. 2010). Covalent binding of alcohol dehydrogenase on attapulgite nanofibers (hydrated magnesium silicate) has been opted owing to its thermal endurance and variable nano sizes (Zhao et al. 2010). Biocatalytic membranes have been useful in unraveling effective covalent interactions with silicon-coated enzymes (Hilal et al. 2006). Cross-linked enzyme aggregates produced by precipitation of enzyme from aqueous solution by addition of organic solvents or ionic polymers have been reported (Sheldon 2011). Different orientations of immobilized enzyme on magnetic nanoclusters obtained by covalent binding have found their applications in pharmaceutical industries owing to their enhanced longevity, operational stability and reusability (Yusdy et al. 2009). Maintaining the structural and functional property of enzymes during immobilization is one of the major roles played by a cross-linking agent. One such agent is glutaraldehyde, popularly used as bifunctional cross-linker, because they are soluble in aqueous solvents and can form stable inter- and intra-subunit covalent bonds.

### Affinity immobilization

Affinity immobilization exploits specificity of enzyme to its support under different physiological conditions. It is achieved by two ways: either the matrix is precoupled to an affinity ligand for target enzyme or the enzyme is conjugated to an entity that develops affinity toward the matrix (Sardar et al. 2000). Affinity adsorbents have also been used for simultaneous purification of enzymes (Ho et al. 2004). Complex affinity supports like alkali stable chitosan-coated porous silica beads and agarose-linked multilayered concanavalin A harbor higher amounts of enzymes which lead to increased stability and efficiency (Shi et al. 2003; Sardar and Gupta 2005). Bioaffinity layering is an improvisation of this technique that exponentially increases enzyme-binding capacity and reusability due to the presence of non-covalent forces such as coulombic, hydrogen bonding, van der Waals forces, etc. (Sardar and Gupta 2005; Haider and Husain 2008).

### Entrapment

Entrapment is caging of enzymes by covalent or non-covalent bonds within gels or fibers (Singh 2009). Efficient encapsulation has been achieved with alginate–gelatin–calcium hybrid carriers that prevented enzyme leakage and provided increased mechanical stability (Shen et al. 2011). Entrapment by nanostructured supports like electrospun nanofibers and pristine materials have revolutionalized the world of enzyme immobilization with their wide-ranging applications in the field of fine chemistry, biomedicine biosensors and biofuels (Dai and Xia 2006; Kim et al. 2006; Wang et al. 2009; Wen et al. 2011). Prevention of friability and leaching and augmentation of entrapment efficiency and enzyme activity by *Candida rugosa* lipase entrapped in chitosan have been reported. This support has also been reported to be non-toxic, biocompatible and amenable to chemical modification and highly affinitive to protein due to its hydrophilic nature (Betigeri and Neau 2002). Entrapment by mesoporous silica is attributed to its high surface area, uniform pore distribution, tunable pore size and high adsorption capacity (Ispas et al. 2009). Simultaneous entrapment of lipase and magnetite nanoparticles with biomimetic silica enhanced its activity in varying silane additives (Chen et al. 2011a). Sol–gel matrices with supramolecular calixarene polymers have been used for entrapment of *C. rugosa* lipase keeping in view their selective binding and carrying capacities (Erdemir and Yilmaz 2011). Lipases entrapped κ-carrageenan has been reported to be highly thermostable and organic solvent tolerant (Tümtürk et al. 2007; Jegannathan et al. 2010).

## Materials used for fabrication of immobilization supports

### Natural polymers as supports

#### Alginate

Alginate derived from cell walls of brown algae are calcium, magnesium and sodium salts of alginic acid and have been extensively used for immobilization as xanthan–alginate beads, alginate–polyacrylamide gels and calcium alginate beads with enhanced enzyme activity and reusability. Cross-linking of alginate with divalent ions (like Ca^2+^) and glutaraldehyde improves the stability of enzymes (Elçin 1995; Flores-Maltos et al. 2011).

#### Chitosan and chitin

Natural polymers like chitin and chitosan have been used as supports for immobilization (Vaillant et al. 2000; Kapoor and Kuhad 2007). The protein or carbohydrate moieties of enzymes are used for binding them to chitosan (Hsieh et al. 2000). Chitosan has been used in combination with alginate where chitosan-coated enzymes had less leaching effect compared to alginate owing to the physical and ionic interactions between the enzyme and support (Betigeri and Neau 2002). Similarly, a wet composite of chitosan and clay proved to be more reliable for enzyme trapping, because it has hydroxyl and amino groups, which easily link with enzymes, together with good hydrophilicity and high porosity. Chitosan in the form of beads can entrap twice as much of the enzymes (Chang and Juang 2007). According to Chern and Chao (2005), the chitin-binding domain of chitinase A1 from *Bacillus circulans* has a high affinity to chitin; so, this property has been exploited to retain D-hydantoinase.

#### Collagen

Being a natural polymer, collagen has been used for immobilization of tannase employing glutaraldehyde as cross-linking agent (Katwa et al. 1981). Fe^3+^-collagen fibers proved to be excellent supporting matrix for catalase immobilization by retaining significant activity even after 26 reuses (Chen et al. 2011b).

#### Carrageenan

Carrageenan, a linear sulfated polysaccharide, has been consistently used for immobilizing a variety of enzymes, like lipase for improving stability (Tümtürk et al. 2007). This support is pseudoplastic in nature, which helps it to thin under shear stress and recover its viscosity once the stress is removed. Jegannathan et al. (2010) could achieve an encapsulation efficiency of 42.6 % by the co-extrusion method using the same support for biodiesel production. Carrageenan has been reported as a cheap and durable support with better entrapment for lactic acid and α-galactosidase enzyme (Rao et al. 2008; Girigowda and Mulimani 2006).

#### Gelatin

Gelatin is a hydrocolloid material, high in amino acids, and can adsorb up to ten times its weight in water. Its indefinite shelf life has attracted attention for enzyme immobilization. Gelatin has been utilized in mixed carrier system with polyacrylamide where cross-linking with chromium (III) acetate proved better than chromium (III) sulfate and potassium chromium (III) sulfate (Emregul et al. 2006). Calcium alginate with gelatin forms a good template for calcium phosphate deposition for enzyme immobilization, and gelatin in combination with polyester films promoted 75 % loading efficiency, compared to previous studies which had 50 % loading efficiency (Shen et al. 2011; Ateş and Doğan 2010).

#### Cellulose

This most abundant natural polymer has been widely used to immobilize fungi laccase, penicillin G acylase, glucoamylase, α-amylase, tyrosinase, lipase and β-galactosidase (Al-Adhami et al. 2002; Mislovicová et al. 2004; Bryjak et al. 2007; Namdeo and Bajpai 2009; Labus et al. 2011; Huang et al. 2011; Klein et al. 2011). Diethylaminoethyl (DEAE)-modified cellulosic supports have longer storage capacity (Al-Adhami et al. 2002). Cellulose-coated magnetite nanoparticles have been used for starch degradation where the attachment of α-amylase to cellulose dialdehyde-coated magnetite nanoparticles resulted in the formation of a novel starch degrading system (Namdeo and Bajpai 2009). Immobilization with ionic liquid-cellulose film activated by glutaraldehyde gave better formability and flexibility (Klein et al. 2011).

#### Starch

Made of linear amylase and branched amylopectin units, starch has been used as enzyme immobilizer. Calcium alginate–starch hybrid supports were applied for surface immobilization and entrapment of bitter gourd peroxidase. Entrapped enzyme was more stable in the presence of denaturants like urea due to internal carbohydrate moieties, while surface-immobilized enzyme had superior activity (Matto and Husain 2009). Radiation grafting of substances like acrylamide and dimethylaminoethyl methacrylate onto starch are among the widely used industrial techniques for a high product yield (Dung et al. 1995; Raafat et al. 2011).

#### Pectin

This structural heteropolysaccharide along with 0.2–0.7 % glycerol acts as plasticizer to reduce brittleness of support and has been used to immobilize papain and for development of new materials for skin injury treatment (Ceniceros et al. 2003). Pectin–chitin and pectin–calcium alginate support have enhanced thermal and denaturant resistance and catalytic properties of entrapped enzymes due to the formation of high stable polyelectrolyte complexes between the enzyme and the pectin-coated support (Gómez et al. 2006; Satar et al. 2008).

#### Sepharose

CNBr-activated Sepharose-4B has been used to immobilize amylase and glucoamylase owing to its porosity and easy adsorption of macromolecules. Further matrix modifications like alkyl substituted Sepharose with multipoint attachment between hydrophobic clusters of the enzyme and alkyl residues of the support play a major role in retaining the catalytic properties at extremes of pH, high salt concentrations and elevated temperatures (Hosseinkhani et al. 2003). Another example of modified Sepharose matrix is concanavalin A (Con A)–Sepharose 4B where biospecific interaction between the glycosyl chains of the enzyme and Con A plays a pivotal role in fabrication of various biosensors (Mirouliaei et al. 2007).

### Synthetic polymers as supports

Ion exchange resins/polymers are insoluble supports with porous surface for enzyme trapping. Amberlite and DEAE cellulose, renewable matrices with large surface area, have been used for immobilization of α-amylase (Kumari and Kayastha 2011). During white radish peroxidase immobilization, glutaraldehyde and polyethylene glycol act as an additive and protective layer around the active center of the enzyme to prevent the attack of free radicals (Ashraf and Husain 2010). Some synthetic polymers used as enzyme supports are stated as follows: polyvinyl chloride that prevents enzyme, cyclodextrin glucosyltransferase from thermal inactivation; polyurethane microparticles derived from polyvinyl alcohol and hexamethyl diisocyanate in the ratio of 1:3 with high enzyme loading and efficiency; UV-curable methacrylated/fumaric acid-modified epoxy that is proposed to be useful for industrial applications; polyaniline in two different forms, viz. emeraldine salt and emeraldine base powder used for covalent binding of α- amylase; glutaraldehyde-activated nylon for immobilizing lipase and UV-activated polyethylene glycol having high porosity employed for wastewater treatment (Abdel-Naby 1999; Kahraman et al. 2007; Pahujani et al. 2008; Romaskevic et al. 2010; Xiangli et al. 2010; Ashly et al. 2011).

### Inorganic materials as supports

#### Zeolites

Zeolites or ‘molecular sieves’ are microporous crystalline solids with well-defined structures and shape-selective properties and are widely used in molecular adsorption. Microporous zeolites were found to be a better support for α-chymotrypsin immobilization than microporous dealuminized ones because of the presence of more hydroxyl groups that form strong hydrogen bonds with the enzyme (Xing et al. 2000). Likewise, Na Y zeolite was used to immobilize lysozyme because it had higher activity compared to other supports as reported by Chang and Chu (2007). The heterogeneous surface of zeolites with multiple adsorption sites are considered to be suitable for modulating the enzyme and support interactions (Serralha et al. 1998).

#### Ceramics

Immobilization of *Candida antarctica* lipase on ceramic membrane showed that this inert support could be exploited for carrying out hydrolytic and synthetic reactions by limiting feedback inhibition (Magnan et al. 2004). Ceramic foams containing both macro (77 nm) and micropores (45 μm) was found to be efficient in lowering diffusion rate and increasing the specific surface area (Huang and Cheng 2008). Another example of ceramics is toyonite whose variable pore structure can be modified using different organic coatings (Kamori et al. 2000).

#### Celite

Celite is highly porous diatomaceous, bioaffinity material and has been used for immobilization of lipase, polyphenol oxidases and β-galactosidase, because it is an inexpensive support having low polarity and large adhesion area (Khan et al. 2006; Liu et al. 2009; Ansari and Husain 2011). It provides resistance against high pH or temperature, urea, detergents and organic solvents (Khan et al. 2006). Celite acts as an additive in sol–gel matrix for ω-transaminases immobilization. It has been preferred due to its chemical inertness and interconnected pore structure (Koszelewski et al. 2010).

#### Silica

Enzymes like lignin peroxidase and horseradish peroxidase (HRP) immobilized on activated silica have been effectively used for the removal of chlorolignins from eucalyptus kraft effluent (Dezott et al. 1995). α-Amylase immobilized on silica nanoparticles improves cleaning performance of detergents. They have been used because of their nano-sized structures with high surface area, ordered arrangement and high stability to chemical and mechanical forces (Soleimani et al. 2011). Surface modifications of silica by amination of hydroxyl and reactive siloxane groups and addition of methyl or polyvinyl alcohol groups strengthen enzyme and support bonds (Rao et al. 2000; Shioji et al. 2003; Pogorilyi et al. 2007).

#### Glass

Glass is a highly viscous liquid and has been employed in immobilizing α-amylase; phthaloyl chloride containing amino group functionalized glass beads was found to be robust and renewable for the process (Kahraman et al. 2007). Another enzyme nitrite reductase was immobilized on controlled pore glass beads, which served as a biosensing device for continuous monitoring (Rosa et al. 2002). Urease immobilized on glass pH-electrodes has provided a stable biosensor for monitoring as low as 52 μg/ml urea in blood samples (Sahney et al. 2005).

#### Activated carbon

Both natural and hydrochloric acid-modified activated carbon has provided valuable support for enzyme adsorption (Alkan et al. 2009). Lately, mesoporous-activated carbon particles containing large contact sites for enzyme immobilization have been used for immobilizing acid protease and acidic lipases where catalytic efficiency has been significantly maintained after 21 cycles of reuse (Kumar et al. 2010; Ramani et al. 2011). It was also found that activated carbon with a high surface area (600–1,000 m^2^ g^−1^) and a significant fraction of its pore volume in the 300–1,000 Å range was suitable for enzyme immobilization (Daoud et al. 2010).

#### Charcoal

Chemical modification of charcoal by adsorbing papain with sulfhydryl groups increased the number of active sites and has been utilized for recovery of mercury from aqueous solution and efficiently employed for industrial wastewater treatment (Dutta et al. 2009). Charcoal supports have been also used in food industries for immobilizing amyloglucosidase for starch hydrolysis without any cross-linking agent and has 90 % catalytic activity (Rani et al. 2000). As reported earlier by Kibarer and Akovali (1996), charcoal is an excellent adsorbent with high adsorptive capacity and minimum fine particulate matter release.

## Applications and scope

Biocatalysts are the key players in various industrial processes. Constant efforts are being made to improve the enzyme’s activity, efficiency, reproducibility and stability during industrial processes (Wang et al. 2010). Production of regioselective and enantioselective compounds for biomedical application has been possible by immobilized enzymes (Ren et al. 2006; Lee et al. 2009). Glucose biosensors have been developed using electrospun PVA and surface-modified carbon nanotubes (Wen et al. 2011). Hydrogen peroxide biosensors have been devised using γ-aluminum trioxide nanoparticles/chitosan film-modified electrode (Liu et al. 2010). Agarose–guar has been successfully utilized for designing phenol biosensors (Bagal and Karve 2006). Currently, keen efforts are being taken for increasing the stability of biosensors. Immobilization of biosensing enzymes into nanocavities showed significant results (Vamvakaki and Chaniotakis 2007). Biosynthesis of polyester has been facilitated by immobilized *C. antarctica* lipase B, a greener alternative to petroleum-based conventional catalysts (Idris and Bukhari 2011). With the advent of nanotechnology, silica nanoparticles with immobilized laccase have been applied for elimination of micropollutants from wastewater (Zimmermann et al. 2011). Increasing environmental concerns have led to the use of immobilized biocatalysts for biodiesel production (Jegannathan et al. 2010).

**Fig. 1:**
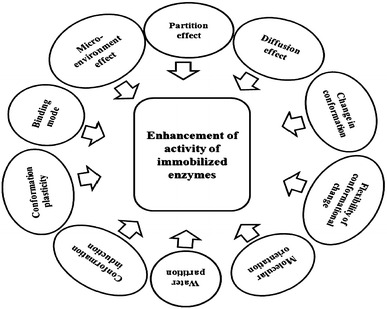
Determinants of enzyme immobilization and activity

The different factors influencing enzyme immobilization and the possible modifications for their enhancement in activity have been chalked out in Fig. 1.

## Conclusion

With the vast array of research on enzyme immobilization, we can conclude that it is one of the most promising techniques for highly efficient and economically competent biotechnological processes in the field of environmental monitoring, biotransformation, diagnostics, pharmaceutical and food industries. Enzyme-based strategies are increasingly replacing conventional chemical methods in both laboratories and industries with attributes like efficiency, quicker performance and multifarious use. However, commercialization of immobilized enzymes is still at a lower pace because of their costs and storage problems. Research should be focused to overcome the current limitations related to immobilization techniques, so as to expand the horizon for all-round application.
